# Exploring the Therapeutic Role of Rab8a in 4R‐Tauopathy

**DOI:** 10.1002/alz.092498

**Published:** 2025-01-03

**Authors:** Mayur S Parmar

**Affiliations:** ^1^ Dr. Kiran C. Patel College of Osteopathic Medicine, Nova Southeastern University, Clearwater, FL USA

## Abstract

**Background:**

Progressive Supranuclear Palsy (PSP) and Corticobasal Degeneration (CBD) are characterized by abnormal aggregation and deposition of tau proteins in neurons and supporting brain cells. The underlying pathophysiology of these 4R‐tauopathy disorders remains unclear. In Alzheimer’s disease (AD), a related tauopathy, vesicle trafficking deficits, and impaired protein clearance are observed early in disease progression. Rab GTPase proteins play an important role in vesicular trafficking, endocytosis, and endosomal‐lysosomal pathways and have been implicated in Parkinson’s disease (PD) and AD pathology. In AD, the normal function of specific Rab proteins is disrupted early and may contribute to abnormal tau accumulation and neurofibrillary tangle formation. Among various Rabs GTPases, Rab8a’s role in AD pathology is explored and shown to accelerate endocytosed Aβ trafficking to the lysosomes and has expression found to decrease in PS1 mutant expressing cells. In contrast, Rab proteins, including Rab8a, and their contribution to 4R tauopathies such as PSP and CBD are little known.

**Method:**

In the postmortem tissues from AD, PSP, and CBD, Rab8a protein expression was determined using western blot analysis. Further, human neuroglioma H4 cells were co‐transfected with wild‐type or mutant Rab8a and 4R0N or self‐aggregating mutant tau[P301L/S320F].

**Result:**

In H4 cells, overexpression of Rab8a resulted in a marked reduction of total and phospho‐tau. The reduction in total and p‐tau was not observed with mutant (non‐functional) Rab8a overexpression. The Rab8a protein expression was found to be decreased in postmortem brain tissues (frontal cortex) from PSP and CBD compared to control tissue.

**Conclusion:**

The study demonstrates the Rab GTPase, Rab8a, expression in neuropathological tissues from 4R‐tauopathy disorders (PSP and CBD) and AD. In addition, the study provides evidence that Rab8a may regulate tau and associated pathology.

